# Unusual presentation of pilomatricoma: A case report and comprehensive review of the literature

**DOI:** 10.1002/ccr3.8285

**Published:** 2023-12-05

**Authors:** Afsaneh Sadeghzadeh Bazargan, Alireza Jafarzadeh, Niloufar Najar Nobari, Sara Dilmaghani, Nasrin Shayanfar

**Affiliations:** ^1^ Department of Dermatology, Rasool Akram Medical Complex Clinical Research Development Center (RCRDC), School of Medicine Iran University of Medical Sciences (IUMS) Tehran Iran; ^2^ Skin and Stem Cell Research Center Tehran University of Medical Sciences Tehran Iran; ^3^ Department of Pathology, Hazret‐e‐Rasoul Hospital Iran University of Medical Sciences Tehran Iran

**Keywords:** adenexal tumor, appendage, case, dermatology, hair follicle tumor, histology, pathology, pilomatricoma, pilomatrixoma, report, report, skin appendage tumor

## Abstract

Pilomatricoma is a benign proliferative lesion of skin appendages that often affects the head, upper limbs, and lower limbs. The clinical appearance of the lesions is that of asymptomatic nodules measuring less than 3 cm. pathologically, these skin lesions show the presence of basaloid cell islands, eosinophilic cytoplasmic cells without nuclei, as well as hemorrhage and calcification. In this study, we present the case of an 8‐year‐old girl with a 5 × 5 cm skin lesion on the forearm, which lacked the typical firmness associated with pilomatricoma lesions during examination. After biopsy, the lesion was confirmed to be pilomatricoma. Furthermore, we have reviewed studies documenting pilomatricoma lesions with atypical clinical features. Based on reports of different clinical manifestations of pilomatricoma in these studies, we suggest that the clinical diagnosis of pilomatricoma should not be limited to the typical presentation of these lesions. In cases where the lesions exceed 3 cm in size, display cystic characteristics, are painful, or resemble keloids, consideration should also be given to the possibility of pilomatricoma.

## Graphical abstract

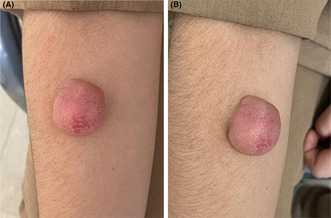

## 1 INTRODUCTION

Pilomatricoma is known as one of the benign tumors of the skin appendages. This tumor originates from the matrix cells of the hair follicle and often affects the neck area in young adults and children.^1^ The clinical appearance of the lesion is a firm and single subcutaneous nodule, which is usually asymptomatic.^2^, ^3^ The prognosis for this tumoral lesion is good, and it is recommended to completely excise the lesion as the treatment.^3^

One diagnostic challenge of this tumor is the possibility of rare variants with different clinical presentations. In these cases, biopsy and histopathological examination are crucial for diagnosis. The pathological appearance of the lesion includes basophilic cells and shadow cells, potentially accompanied by calcification, hemorrhage, and variable lymphocyte infiltration.^4^

In this study, we present the case of an 8‐year‐old girl with a large and unusual nodular lesion on her forearm. The lesion had been gradually growing for 6 months prior to her visit. We discuss cases of these atypical manifestations of pilomatricoma.

## 2 CASE PRESENTATION

**FIGURE 1:**
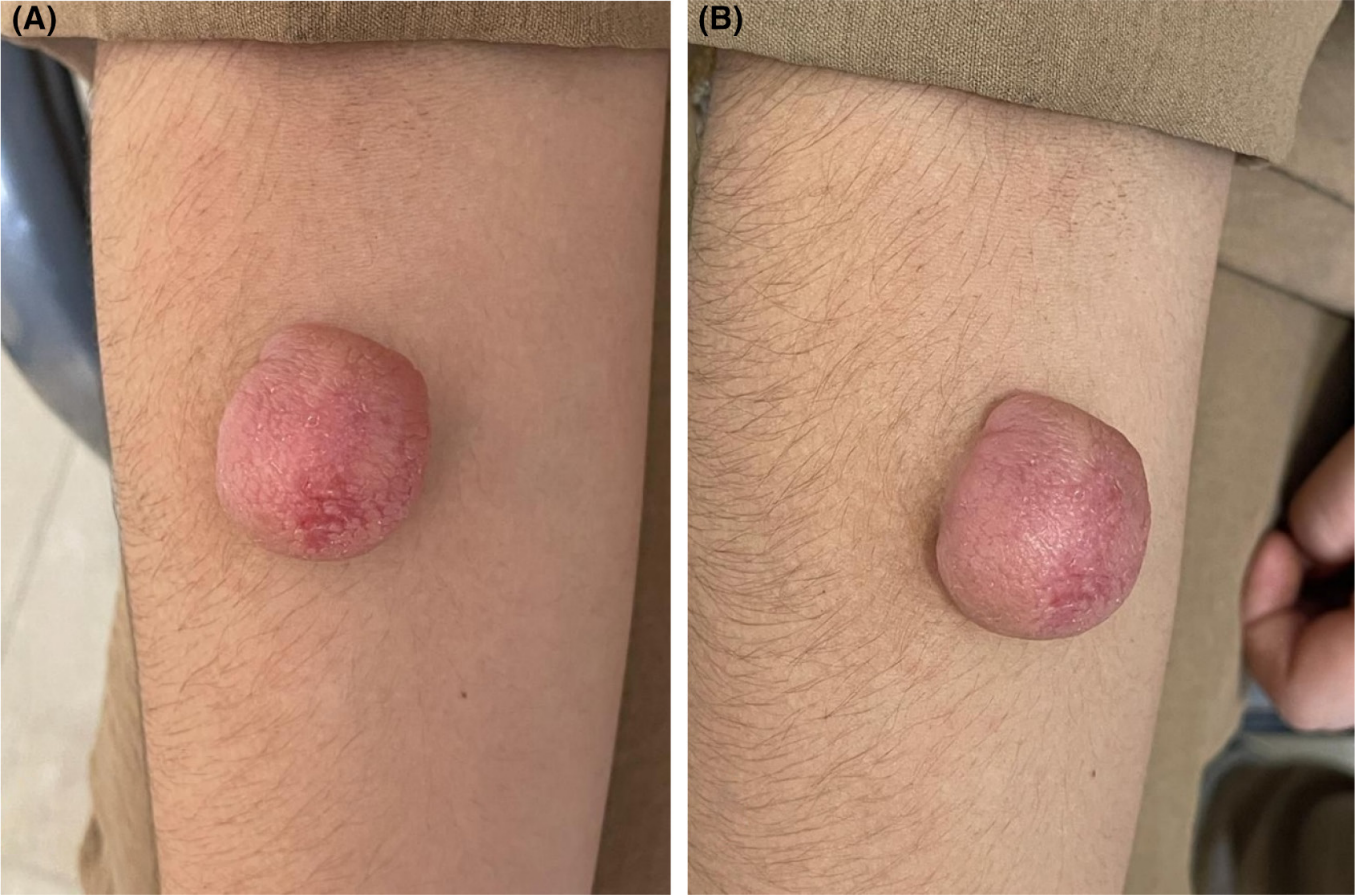
(A, B) Pilomatricoma lesion in the forearm of an 8‐year‐old patient.

The patient we studied was an 8‐year‐old girl who complained of a proliferative lesion about 6 months ago. Initially, it presented as a papule and gradually increased in size (Figure 1). The size of the lesion was 5 × 5 cm, and the lesion was asymptomatic, with no reports of itching or pain. During the examination, the consistency of the lesion was found to be soft and not multilobular. There was no tenderness observed in the lesion. The supplementary history did not mention any history of trauma or inflammation in the area. No other pathological lesions were found on other areas of her skin. Additionally, there is no history of any systemic diseases or recent surgery. In order to diagnose the patient, an incisional biopsy of the lesion was performed.

**FIGURE 2:**
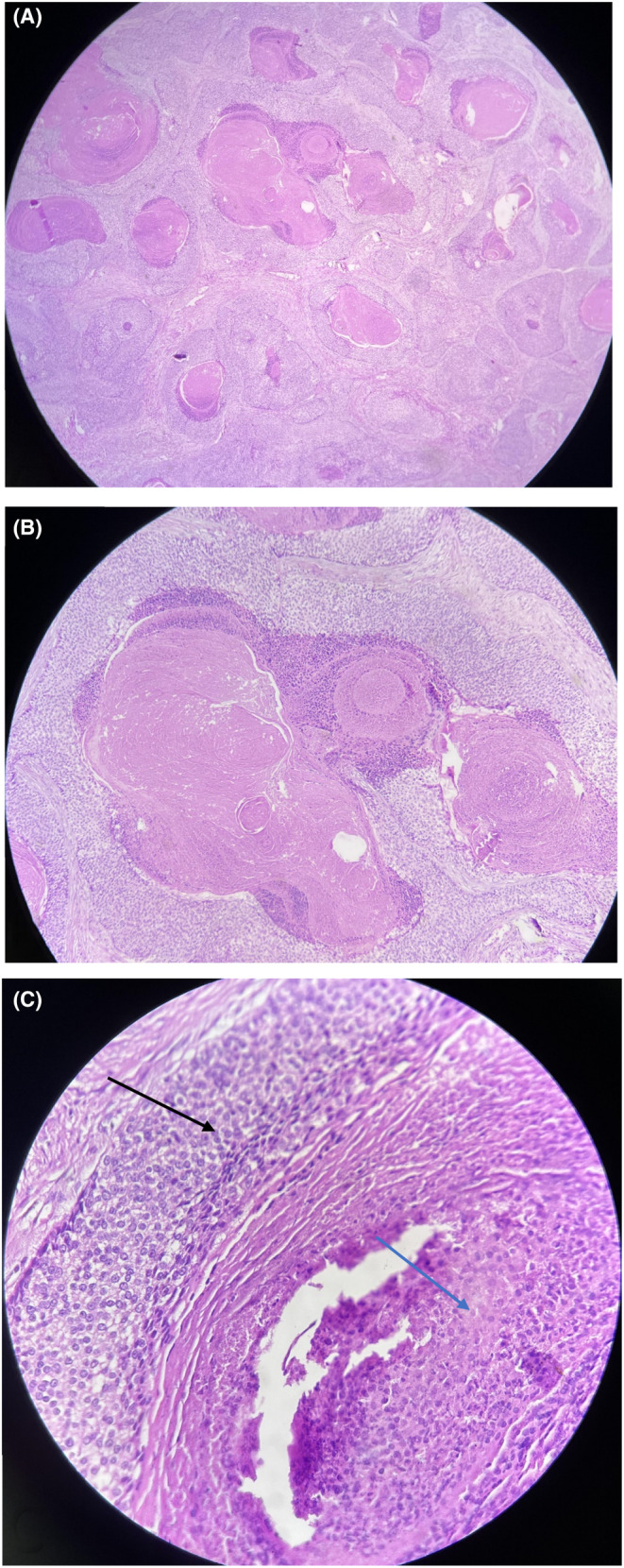
(A, B, C) Pathological view of a pilomatricoma lesion reveals basaloid cells (black arrow) and eosinophilic cell structures without nuclei (also known as shadow cells) (blue arrow).

The pathologist's report showed the accumulation of basaloid cells around the cell structures with eosinophilic cytoplasm and without a nucleus (shadow cells) in the reticular dermis (Figure 2). Based on these findings, the diagnosis of pilomatricoma was proposed for her.

After confirming the diagnosis, the patient underwent excision of the lesion, and there was no recurrence during the 6‐month period following the surgery.

## 3 DISCUSSION

Pilomatricoma is a benign skin tumor that is commonly found on the head, neck, and upper limbs, especially among children and adolescents.^5^ It represents approximately 1% of all benign skin lesions and typically carries a favorable prognosis.^1^ The origin of the lesion is the matrix of the hair follicle, and it is more prevalent in women. The size of the lesions is typically less than 3 cm.^6^, ^7^

Despite the usual manifestation of this skin tumor being a single, hard, asymptomatic lesion, previous studies, as well as our current study, have reported unusual manifestations of this skin lesion.^6^, ^8^, ^9^, ^10^ Osorio et al.^8^ reported a case of pilomatricoma occurring in the orbital cavity of a 41‐year‐old man, measuring 1 × 1 cm in size. Although the consistency of the lesion was firm, typical of pilomatricoma, the location and occurrence in middle age were unusual.

In another study by Belliappa et al.,^6^ a 17‐year‐old girl presented with a bolus lesion of pilomatricoma. The lesion appeared as a 2 cm long blister on an erythema base, which had been present on her arm for the past 3 months. This study is consistent with our findings in terms of the non‐firmness of the lesion and the location, but our patient did not exhibit blistering.

The two studies mentioned above did not show any unusual characteristics in terms of lesion size. In fact, the lesions in those studies were smaller compared to the ones observed in our study. However, Hawkes et al.^10^ reported a case of pilomatricoma with dimensions measuring 8 × 9 cm. This particular lesion appeared as a solitary, red and firm mass on the chest of a woman. It had gradually grown over a period of 6 years. Another case of giant pilomatricoma was documented by Marzouki et al.^11^ In this case, a 32‐year‐old woman had a mass measuring 6 cm × 6 cm on her arm. The lesion was firm yet movable, and it was successfully treated with excisional surgery. Although the size of these lesions more closely resembles our own case, it is important to note that the consistency of the lesions in our study is soft.

The presence of multiple clinical manifestations of pilomatricoma lesions necessitates a biopsy of the lesions and their histopathological examination. The pathological appearance of these lesions is characterized by a combination of basaloid cells and cells with eosinophilic cytoplasm (known as shadow cells). Additionally, calcification and hemorrhage often occur.^7^

Several differential diagnoses can be proposed for pilomatricoma lesions, including trichilemmal cyst, basal cell carcinoma, keratoacanthoma, squamous cell carcinoma, hemangioma, metastasis, and other benign tumors of skin appendages such as cylindroma and spiradenoma.^7^, ^8^ It is worth mentioning that ulcerative and draining lesions can be included in the differential diagnoses of pilomatricoma. In fact, Mohr et al published a report in 2023 regarding a case of a 4‐year‐old child with an ulcerative and draining lesion. The biopsy and histopathological examination revealed a rare type of pilomatricoma known as perforating pilomatricoma.

Malignancy in pilomatricoma lesions is rare, and there is no evidence of malignancy reported in our patient. However, Nishioka et al.^12^ published a report of a case of pilomatrix carcinoma in a 38‐year‐old woman who had a history of a pilomatrix tumor at the back of her neck for 10 years. The tumor had increased in size over the past 3 months. Upon pathological examination of this patient, atypical cells, increased mitosis, and necrosis were observed, indicating evidence of malignancy.

Suggested treatments for pilomatricoma include excision of the lesion, punch incision, and curettage. In the case of malignant pilomatricoma lesions, it is recommended to remove the lesion with a margin of 0.5 to 1 cm.

In the end, we summarized the studies investigating the atypical manifestations of pilomatricoma in a table and compared them from various perspectives (Table 1).

**TABLE 1:** Comparison of studies reporting unusual manifestations of pilomatricoma (since 2010).

Variables	Our study	Osorio et al.^8^	Mohr et al.^14^	Belliapa et al.^6^	Hawkes et al.^10^	Kapoor et al.^13^
Gender	Female	Male	Male	Female	Female	Male
Age(years)	8	41	4	17	unknown	48
Unusual finding	Lack of firm consistency, pedunculated mass, larger size than 3 cm.	Unusual location	Ulcerative and draining lesion	semi‐transparent	Ulcerative lesion, larger size than 3 cm and unusual location	Unusual location
				Blister		
location of the lesion	Forearm	Orbit	Neck	Arm	Chest	Breast

Variables	Marzouki et al.^11^	Sung et al^16^	Shetty et al^2^	Ingaki et al^1^	Dewi et al^15^
Gender	Female	Female	Male	Female	Female
Age(years)	32	5	60	52	9
Unusual finding	Larger size than 3 cm.	Fluctuating, cystic mass	Ulcerated surface, larger size than 3 cm	Painful cystic lesion in an unusual location.	Unusual location and mild tenderness
Location of the lesion	Arm	Arm	sternum	Ear	Ear

## 4 CONCLUSION

We have concluded that when diagnosing pilomatricoma lesions, it is important to consider rare and less common variants. This skin tumor may not always present as a small, hard, and asymptomatic lesion in the head and neck region. Instead, the presence of cystic, soft, painful, ulcerous, or pseudo‐keloid lesions larger than 3 cm may also indicate pilomatricoma. In such cases, the pathological examination of the lesions will help confirm the diagnosis. We recommend that future studies report any unusual manifestations of this skin tumor to ensure that its diagnosis is not overlooked.

## AUTHOR CONTRIBUTIONS

**Afsaneh Sadeghzadeh Bazargan:** Methodology; project administration; writing – review and editing. **Alireza Jafarzadeh:** Conceptualization; supervision; writing – original draft. **Niloufar Najar Nobari:** Data curation; writing – original draft. **Sara Dilmaghani:** Formal analysis; validation. **Nasrin Shayanfar:** Investigation; software; writing – original draft.

## FUNDING INFORMATION

None.

## CONFLICT OF INTEREST STATEMENT

The authors have no conflict of interest to declare.

## CONSENT STATEMENT

After providing the necessary explanations, written informed consent was obtained from the patient regarding the submission of their clinical condition to medical journals. Additionally, the patient has been assured that their name and personal details will be kept confidential by the authors.

## ETHICAL APPROVAL

The researchers were committed and adhered to the principles of the Helsinki Convention and the Ethics Committee of the Iran University of Medical Sciences in all stages.

## TRANSPARENCY DECLARATION

Authors declare that the manuscript is an honest, accurate, and transparent. No important aspect of the study is omitted.

## Data Availability

All data produced in the present study are available upon reasonable request to the authors.
